# The mechanics of brittle granular materials with coevolving grain size and shape

**DOI:** 10.1098/rspa.2020.1005

**Published:** 2021-05

**Authors:** Giuseppe Buscarnera, Itai Einav

**Affiliations:** ^1^ Department of Civil and Environmental Engineering, Northwestern University, Evanston, IL, USA; ^2^ School of Civil Engineering, The University of Sydney, Sydney 2006, Australia

**Keywords:** granular materials, comminution, grain shape

## Abstract

The influence of particle shape on the mechanics of sand is widely recognized, especially in mineral processing and geomechanics. However, most existing continuum theories for engineering applications do not encompass the morphology of the grains and its evolution during comminution. Similarly, the relatively few engineering models accounting for grain-scale processes tend to idealize particles as spheres, with their diameters considered as the primary and sole geometric descriptor. This paper inspires a new generation of constitutive laws for crushable granular continua with arbitrary, yet evolving, particle morphology. We explore the idea of introducing multiple grain shape descriptors into Continuum Breakage Mechanics (CBM), a theory originally designed to track changes in particle size distributions during confined comminution. We incorporate the influence of these descriptors on the elastic strain energy potential and treat them as dissipative state variables. In analogy with the original CBM, and in light of evidence from extreme fragmentation in nature, the evolution of the additional shape descriptors is postulated to converge towards an attractor. Comparisons with laboratory experiments, discrete element analyses and particle-scale fracture models illustrate the encouraging performance of the theory. The theory provides insights into the feedback among particle shape, compressive yielding and inelastic deformation in crushable granular continua. These results inspire new questions that should guide future research into crushable granular systems using particle-scale imaging and computations.

## 1. Introduction

The significance of particle shape in geosciences, geotechnology and mineral processing is widely recognized [1,2]. The particle shape influences the efficiency with which sand assemblies store and dissipate energy, as well as their flowability and strength. Grain morphology also affects the state of bulk density in granular systems, as it impacts how particles slip, rotate and rearrange under arbitrary stresses [3,4]. In return, this impacts local and collective processes ranging from contact deformation and grain fracture to volume change and critical density-stress state relationships [5–7]. Novel digital imaging techniques have greatly expanded our ability to examine initial and evolving particle shapes, with major benefits for the in situ characterization of continuum-scale processes [8–10]. Similarly, computational advances have allowed us to replicate complex particle shapes through geometric analogues ranging from sphere clumps [11] to polyhedra [12], ellipsoids [13] or level sets [14], with corresponding benefits for the study of particle fracture and shape evolution [15–17]. Nevertheless, despite the increasing availability of powerful characterization and simulation tools, these alone cannot provide a clear path into quantifying the engineering effects of grain shape on the macroscopic constitutive response of the material.

This challenge is epitomized by the laboratory scale assessment of the elastic properties of sand. An example is the work of Cho *et al*. [18], who tested materials characterized by a wide range of particle shapes, classified according to standard descriptors such as sphericity and roundness. One of the key conclusions of their effort was that, among other effects, the geometric irregularity of sand grains reduces the small strain shear stiffness of the material and increases its pressure sensitivity. Later contributions, however, presented remarkably different trends. For example, experiments with mixtures of grains with different shapes found that an increase in the fraction of angular particles actually increases the small strain shear stiffness of the assembly [19]. Similarly, Altuhafi *et al.* [20] examined a large database of sands through a compounded descriptor of overall particle irregularity. Their analysis also showed that as grains are less spherical, the rate of increase of their normalized small strain shear stiffness with pressure is intensified. Such seemingly conflicting findings are rooted in the experimental difficulty of isolating the influence of the particle shape, which without proper data treatment may be overshadowed by concurrent effects due to different initial packing, fabric and size polydispersity. In this context, an enlightening perspective was offered by the careful study of Liu & Yang [21], who used well-controlled experiments to identify the effect of porosity, gradation and particle shape in the measurement of the small strain stiffness. Contrary to common expectations, and in light of the same standard descriptors used by Cho *et al*. [18], it was shown that samples made of round, spherical particles actually display lower stiffness when compared with those consisting of more irregular grains, as long as all other factors were kept equal.

The challenges listed above are not specific to elastic properties. There is indeed abundant evidence for a feedback between particle-shape evolution and yielding in compressed sands, which are heavily influenced by grain fracture [22]. There is also substantial consensus that grain shape influences the crushing strength of both individual particles and packed assemblies, including data that show decreasing compressive yield strength with increasing particle irregularity [23–25]. In fact, grain irregularities are often expected to expedite the onset of crushing along with relatively slow morphological changes upon compression. Conversely, round particles were found to yield under higher pressures, thus displaying more intense and rapid shape alterations once the crushing threshold is overcome. Despite these results, no definitive insight has yet been given on whether these trends emerge from local effects (e.g. contact indentation) or collective behaviours (e.g. fabric and particle coordination) modulated by the shape of the grains.

**Figure 1.**
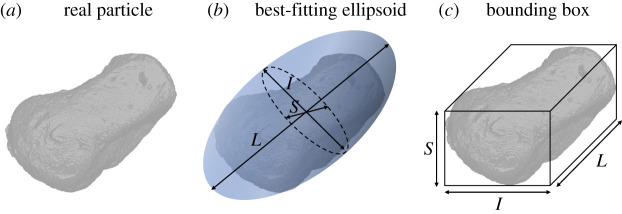
Simplified representation of particle shape in three dimensions: (*a*) sand grain scanned through X-ray tomography [28]; (*b*) approximation based on outer fitting ellipsoid; (*c*) prismatic approximation based on bounding box. (Online version in colour.)

In this paper, we argue that the most significant obstacle hindering the interpretation of measurements involving different particle shapes is the lack of a rigorous framework explaining why a departure from perfect particle sphericity influences the storage of elastic strain energy and its subsequent release upon grain crushing. To fill this gap, we propose a new constitutive theory for crushable granular materials that captures the effect of initial non-spherical particle shapes on both elastic deformation and yielding, as well as the evolution of the shape of the particles upon compression. For this purpose, we generalize the structure of Continuum Breakage Mechanics (CBM) [?] by incorporating the effects of the particle shape, on top of the primary consideration of grain size effects in that theory. From this standpoint, we give to the term *shape* a specific connotation. In particular, we regard the shape of sand particles as a specific category belonging to the general notion of particle *morphology* [27]. Here, morphology is seen as the ensemble of irregularities that differentiate a grain from a spherical object at all length scales, thus including irregularities as large as the particle itself (i.e. those determining aspect ratio and elongation), as well as features orders of magnitude smaller than the nominal particle size, such as those defining its roundness or smoothness. By contrast, we employ the term *shape* to refer to the coarsest class of irregularities, i.e. those manifesting as differences between the principal dimensions of a three-dimensional object and causing a first-order departure from the geometry of a sphere. Possible shape descriptors are given in figure 1 with reference to the fitting of either an enclosing ellipsoid or a bounding prismatic box of a sand grain. In the following sections, we will further consider the effects and evolution of this class of irregularities upon successive grain fracture events.

## 2. Grain shape attractor

Insight about the evolution of grain size and shape is essential to rationalize their influence on the mechanics of sand during comminution. In this context, changes in shape can hardly be decoupled from the microscopic fracture events responsible for grain size reduction. Such geometric descriptors are thus inevitably bound to evolve in conjunction, bearing similarities with biological processes where a species may coevolve whenever another one closely related with it does. This section examines the coevolution of the shape and size of grains in particulate systems as they undergo intense crushing in either laboratory or natural settings, which consistently reveal an almost unique set of geometrical properties. Considerable insight comes from geophysical observations, where even in remarkably complex settings nature displays encouraging signs of order, including regular patterns that emerge even under seemingly chaotic conditions. The phenomenon of particle crushing is no exception. A stunning example of regularity is the size distribution of the particles found in many heavily crushed zones on Earth, such as within active fault gauges [29,30]. Under such dense and almost indefinite shear deformation settings, particulate media approach an ultimate state at which the particle sizes follow a power law distribution that often reflects a self-similar topology. It is this regularity that led to a reappraisal of the origin of sand comminution [22], as well as to recast under a new light the continuum models simulating it [26].

**Figure 2.**
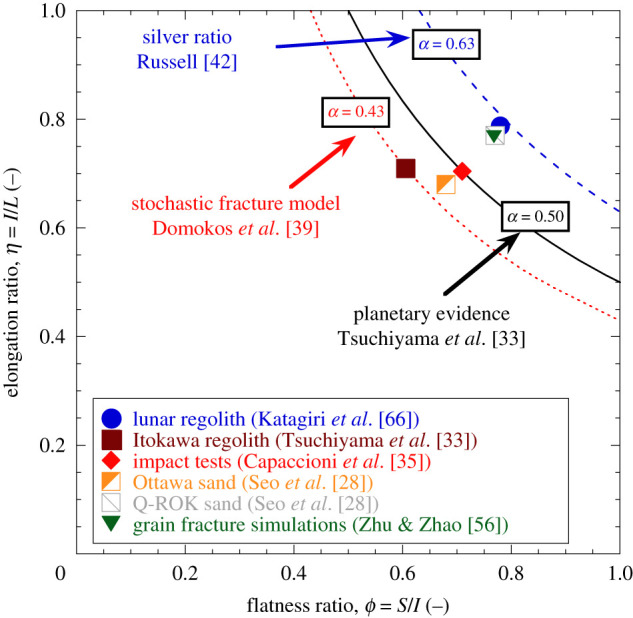
Shape proportions for heavily fragmented particles from natural systems and laboratory experiments. Crushed fragments are represented on the Zingg plane (flatness, *ϕ* = *S*/*I*, against elongation, *η* = *I*/*L*, ratios). The diagram also presents lines of constant equal aspect ratios, *α* = *S*/*L* = *ϕη*, along with a few ultimate ratios reported from the literature. (Online version in colour.)

**Figure 3.**
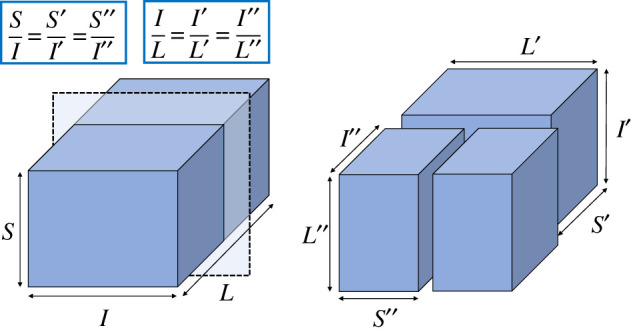
Graphical representation of the concept of silver ratio in three dimensions in relation to shape self-similarity upon successive breakage events. Cutting planes orthogonal to the current longest axis indicate the location of the fracture generating new fragments possessing self-similar geometry. (Online version in colour.)

Similarly, evidence of ultimate shape distributions in fragmented systems is rapidly emerging in the scientific literature, often outside the realm of granular mechanics [31]. Considering such an attractor in terms of particle shape can greatly assist the understanding of how granular systems deform, crush and behave inelastically. Systematic examples of the regular morphology of the products of extreme fragmentation have been reported in planetary systems subjected to impacts [32,33]. By approximating the morphology of the building blocks of these systems as ellipsoids (figure 1), the ratio between the principal dimensions of the fragments found in extraterrestrial impact sites (i.e. *S*, *I* and *L*, respectively, the smallest, intermediate and longest axes) were found in the proportion of $1:\sqrt{2}:2$, corresponding to an aspect ratio *α* = *S*/*L* = 0.5 (figure 2). Remarkably, this finding was insensitive to the size of the fragments—the same ratio applied to boulders making up entire asteroids (i.e. metre-scale objects [32]), as well as to the regolith of these same asteroids’ surfaces or of other celestial bodies (i.e. micrometre-scale particles [33,34]). Evidence of regularity was also found in laboratory experiments mimicking the impacts from which extraterrestrial regoliths originate [35], as well as terrestrial settings where fragmentation emerges from weathering, desiccation, and compression [36–38]. Interpretations of these trends could be made through a stochastic model simulating sequential binary break-ups of polyhedral particles [39]. By optimizing the location and direction of the break-up planes, this model was able to show that above a characteristic size the overall shape of the fragments is well approximated by prisms conforming to a geometric proportion of 1:1.52:2.32 (*α* = *S*/*L* ≈ 0.43), thus reinforcing the idea of a natural tendency of crushed fragments to converge to a uniquely defined anisotropic form. By restricting the discussion to rectangular prisms breaking exclusively at the centre of their longest edge, the same logic can be used to recover the notable geometric proportion referred to as *silver ratio* (figure 3). This hypothesis is particularly appealing in light of the well-known results of fracture mechanics, which identifies the centre of a particle as the location of maximum tensile stress and consequent crack nucleation [40,41]. This term is used in a two-dimensional context to indicate rectangles that maintain their aspect ratio of $1:\sqrt{2}$ unaltered when cut at the centre of their longest side [42]. Interestingly, two-dimensional discrete element method (DEM) models of crushable agglomerates suggest that such a ratio can be a good approximation of the average shape of crushed particles resulting from confined compression [43]. Under three-dimensional conditions, the idea of silver ratio can be generalized through a prism with edges satisfying the proportion $1:\sqrt[{3}]{2}:\sqrt[{3}]{2^{2}}$ (*α* = *S*/*L* ≈ 0.63). In this case, systematic break-up across the longest edge of a fragment generates prisms that always maintain geometrical proportions, thus providing a useful conceptual model for a scenario in which size reduction occurs while preserving shape self-similarity among parent and child grains. These examples of geometric proportions reflect a certain regularity of how the shape of crushed particles evolves. Most notably, they satisfactorily encompass particle crushing data from extremely diverse settings, thus strongly suggesting that the ultimate value of simple shape descriptors for broken solids varies within a relatively narrow, yet consistent range (figure 2).

Here, we use the idea of a shape attractor as a working hypothesis to set up a continuum theory enabling the inspection, simulation and quantification of the role of the particle shape on the macroscale properties of crushable granular solids. While we make no specific decision about the exact numerical value of this attractor, nor about the underlying microscopic mechanisms that produce it, we will use this idea of ultimate conditions in an averaged form relevant for homogenized continua. The hypothesis regarding the existence of a shape attractor (which is well supported by the narrow data scatter in figure 2), along with the use of scaling relations for the elastic properties of granular media [26], will be the key building blocks of an extended CBM framework able to track the coevolution of grain shape and size during comminution.

The reminder of the paper is structured as follows. First, we provide an overview of the original CBM framework for confined comminution. Afterwards, we propose a general strategy to incorporate shape descriptors into CBM. We then specialize this framework to two cases: (i) a single shape descriptor (the particle aspect ratio) and (ii) multiple shape descriptors (here referred to as flatness and elongation ratios). Finally, we discuss open challenges and future research needs related to the study of evolving particle shapes in granular systems.

## 3. Breakage mechanics with spherical particles

This section briefly reviews the key governing equations of CBM while highlighting its structure and key hypotheses. Since most of the fundamental premises of CBM will be retained in its extended formulation for non-spherical particles, this section serves as a basis for the mathematical developments outlined in the subsequent sections. For simplicity, but with no loss of generality, materials in which breakage is the only form of dissipation are considered (i.e. energy loss due to plastic deformations from friction and volumetric grain rearrangement is ignored). Furthermore, the equations are here limited to isotropic stress states, for which we can present the analysis in scalar terms. The removal of these simplifications is straightforward and is extensively elaborated in prior works [26,44,45].

Let *x* be a particle size fraction in a polydisperse material. In this context, the term *size* reflects the volume-equivalent spherical diameter of an arbitrarily shaped grain or any other correlated measure of size (e.g. its largest dimension). In the original CBM, no other particle descriptors are used and the size is treated as an aleatory variable characterized by a probability density function, *p*(*x*), associated with the grain size distribution (GSD) of the continuum. As a consequence, the average grain size can be expressed as:

3.1 ⟨x⟩≡∫p(x)x dx,

where *p*(*x*) satisfies ∫p(x) dx=1. This statistical averaging can be applied to any function dependent on the grain size. For this paper, the most relevant statistical homogenization procedure involves the Helmholtz free energy potential, Ψ, i.e. the thermodynamic function capturing the elastic strain energy storage. The statistical average of Ψ can thus be written as:

3.2 Ψ≡⟨ψ⟩=∫ψ(x)p(x) dx.

In the above equation, the non-homogenized Helmholtz free energy is given by a multiplicative decomposition, *ψ*(*ϵ*, *x*) = *ψ*_*r*_(*ϵ*)*f*_*x*_(*x*), where *ψ*_*r*_ is the free energy potential associated with a reference grain size, *x*_*r*_, and *f*_*x*_ is an energy split function reflecting how the energy is allocated across different fractions. Micromechanical arguments suggest that a power law is the optimal form for *f*_*x*_, as follows:

3.3 $$f_{x}(x)={\left(\frac{x}{x_{r}}\right)}^{n},$$

in which *n* = 2 for spheres. The rationale for such power law scaling is that the strain energy stored in each grain fraction is proportional to the surface area, as larger grains attract a higher number of contact forces, and consequently storing more energy [26]. Another essential feature of CBM is the existence of an ultimate state of the GSD at which confined compression no longer generates crushing. Such hypothesis is reflected by a breakage state variable, *B*, varying from *B* = 0 (unbroken state) to *B* = 1 (complete breakage). Through this state variable it is possible to capture changes in grain size polydispersity, as follows:

3.4 $$p(x,B)=p_{0}(x)(1-B)+p_{u}(x)B,$$

where *p*_0_(*x*) is the initial GSD and *p*_*u*_(*x*) is the ultimate GSD, which is often assumed to be fractal [29]. From this point of view, *B* = 1 acts as an attractor for a crushable material, in that it sets the ultimate state at which no further changes of GSD are possible. The two hypotheses of CBM discussed above can be used to construct a breakage-dependent Helmholtz free energy potential simply by inserting equation (3.4) into (3.2), which leads to

3.5 $$\Psi (\epsilon ,B)=f_{b}(B)\psi_{r}(\epsilon ),\;f_{b}(B)=(1-\theta B),$$

where *θ* is a grading index dependent on *p*_0_ and *p*_*u*_, as well as on the energy split function. Here, the breakage dependence of the function *f*_*b*_ arises due to the statistical homogenization and the scaling of energy with respect to grain size *x*. The above information becomes useful by employing the first and second principles of thermodynamics:

3.6a σ dϵ=dΨ+dΦ

and

3.6b $$\text{d}\Phi =E_{B}\;\text{d}B>0,$$

where *ϵ* is the strain and *σ* is the corresponding work-conjugate stress. As mentioned earlier, here these measures are taken as scalars, to help transparency and simplifying the discussion. Generalization into tensorial formalism is straightforward, which would follow directly from most other earlier papers on CBM, but will not alter the conceptual conclusions of this paper. The term dΦ indicates the breakage dissipation increment, which is equal to the product of the breakage increment, d*B*, and its conjugate thermodynamic force, the breakage energy, *E*_*B*_. Given the additional simplification in this paper, where we neglect any other sources of dissipation additional to the breakage dissipation, no difference shall be assumed hereafter between the elastic strain *ϵ*^*e*^ and the total strain, *ϵ*. Hence, standard thermodynamic formalism implies that:

3.7a $$\sigma =\frac{\partial \Psi }{\partial \epsilon }=f_{b}\frac{\partial \psi_{r}}{\partial \epsilon }$$

and

3.7b $$E_{B}=-\frac{\partial \Psi }{\partial B}=-\frac{\partial f_{b}}{\partial B}\psi_{r}.$$

Appropriate choices for the dissipation function [26,46] lead to the following breakage yielding condition:

3.8 $$y=\frac{E_{B}}{E_{c}}{\left(1-B\right)}^{2}-1<0,$$

through which the following breakage evolution rule and dissipation increment follow:

3.9a $$\text{d}B=2\Lambda \frac{{\left(1-B\right)}^{2}}{E_{c}}$$

and

3.9b $$\text{d}\Phi =2\Lambda \frac{E_{B}{\left(1-B\right)}^{2}}{E_{c}},$$

where *E*_*c*_ is the energy threshold associated with first breakage and Λ is a non-negative inelastic multiplier. From equation (3.8), it is readily apparent that CBM describes yielding in energy terms, thus establishing an analogy with fracture mechanics [47–49]. Furthermore, by linking equation (3.8) with a specific form of Helmholtz free energy it is possible to recover the stress at the onset of breakage. In the case of a quadratic Helmholtz free energy for a reference system constituted solely by initial grain sizes (i.e. linear elasticity prior to breakage):

3.10 $$\psi_{r}=\frac{1}{2}K\epsilon^{2},$$

it follows that the yielding stress is

3.11 $$p_{c}=p_{c}^{L}\frac{f_{b}}{\left(1-B\right)}=p_{c}^{L}\frac{\left(1-\theta B\right)}{\left(1-B\right)},$$

where

3.12 $$p_{c}^{L}={\left(\frac{2KE_{c}}{\theta }\right)}^{1/2}$$

is the initial yielding stress (i.e. *p*_*c*_ at *B* = 0). Since this value is obtained for idealized particles without shape descriptors other than their size, it will be regarded as the reference value for spherical grains. Such value depends on the bulk modulus (through *K*), the macroscopic crushability (through *E*_*c*_) and the proximity between initial and ultimate GSD (through *θ*).

A different expression of *ψ*_*r*_ can be used to recover the nonlinear, pressure-dependent behaviour typical of granular materials. For example, using the development of [44,46] we can use

3.13 $$\psi_{r}=\frac{p_{r}}{\bar{K}(2-m)}{\left(\bar{K}(1-m)\epsilon_{v}+1\right)}^{\left[(2-m)/(1-m)\right]}.$$

In particular, for a constant $m=\frac{1}{2}$

3.14 $$\psi_{r}=\frac{2p_{r}}{3\bar{K}}{\left(\frac{1}{2}\bar{K}\epsilon_{v}+1\right)}^{3},$$

which reflects pressure-dependence due to conical inter-particle contacts [50], thus leading to the following expression of yielding stress:

3.15 $$p_{c}=p_{c}^{N}\frac{f_{b}}{{\left(1-B\right)}^{4/3}}=p_{c}^{N}\frac{\left(1-\theta B\right)}{{\left(1-B\right)}^{4/3}},$$

where

3.16 $$p_{c}^{N}=p_{r}{\left(\frac{3\bar{K}E_{c}}{2\theta p_{r}}\right)}^{2/3},$$

is the initial yielding stress prior to breakage for an assembly of spherical particles, $\bar{K}$ is a non-dimensional stiffness constant, and *p*_*r*_ a reference pressure.

Note that it follows from both equations (3.11) and (3.15) that the crushing strength grows with *B*, a result which underpins the process often referred to as clastic hardening [51].

## 4. Breakage mechanics with non-spherical particles

Here CBM is generalized to continua consisting of non-spherical particles with coevolving grain sizes and shapes. For this purpose, the original CBM formalism is augmented to encompass an arbitrary number, *n*, of shape descriptors, *s*_*i*_. As mentioned previously, while the assessment of the overall particle morphology involves multiple length scales, here the term *shape* refers only to the coarsest set of geometric irregularities, which reflect differences between the principal dimensions of a three-dimensional object (figure 1). In addition, to recover spherical particles as a particular case, the derivation will make reference to ellipsoidal particles, and will focus on the effect of shape on surface area, from which we will be able to estimate the dependence of elastic properties on shape. While the paper has no aim to examine the evolution of morphological descriptors reflecting lower-scale attributes (e.g. roundness and roughness), it is worth remarking that the formalism developed hereafter is general enough to accommodate future extensions.

The first key element of the proposed augmented CBM is the incorporation of the shape descriptors into the Helmholtz free energy potential. In line with the philosophy of the original CBM formulation, this step is pursued by adopting an extended multiplicative decomposition for the non-homogenized free energy function of a given class of grain sizes and shapes:

4.1 $$\psi (\epsilon ,x,s_{1},s_{2},\ldots ,s_{n})=\psi_{r}(\epsilon )\;f_{x}(x)\;f_{s}(s_{1},s_{2},\ldots ,s_{n}),$$

where *ψ*_*r*_ is the free energy for reference-sized and -spherical grains; while *f*_*x*_ and *f*_*s*_ are the energy split functions that come to correct that energy for any grain size *x* and shape {*s*_1_, *s*_2_, …, *s*_*n*_}, respectively. Although the attributes of the *f*_*s*_ function will be later illustrated with reference to specific examples, the logic through which it will be constructed is similar to the rationale outlined in the previous section for *f*_*x*_. Specifically, the possible expressions for *f*_*s*_ will come to describe the effect of shape irregularities on the surface area of a particle. Hence, the value of the reference shape descriptors, $s_{r}^{i}$, will be chosen to ensure that *f*_*s*_ = 1 for the particular case of a sphere. Starting from equation (4.1), statistical homogenization provides a Helmholtz free energy function for the continuum. While this step can be conducted in terms of both size, *x*, and shape descriptors, *s*_*i*_, in a form similar to that illustrated in equation (3.1), here only the grain size will be treated as an aleatory variable with its own probability density function, from which it follows:

4.2 Ψ(ϵ,B,s1,s2,…,sn)=ψr(ϵ) fb(B) fs(s1,s2,…,sn),

where *f*_*b*_ = 1 − *θB* remains as specified in equation (3.5), while the proposition above is boxed as it provides the first key ingredient in the new theory. Specifically, we consider the scaling function *f*_*s*_ to denote the dependence of the Helmholtz free energy on grain shape due to surface area variations. This is consistent with the role of the function *f*_*b*_ that was already used in CBM to denote such surface variations as a function of the GSD. As will be shown in the following, the new factor *f*_*s*_ will enable us to reason changes in elastic properties with grain shape, so much as *f*_*b*_ explains such changes with GSD.

Also note that in equation (4.2) the average value of the shape descriptors is directly used, rather than evaluating it using their potentially evolving statistical distributions. This choice simplifies the derivations and is justified by the scarcity of data of statistical distributions of the shape descriptors. It must be noted, however, that should this type of data become more commonly available, generalizations including such evolving distributions are readily possible simply by following the philosophy of CBM as originally carried out for distributed grain sizes.

The statistically homogenized free energy Ψ in equation (4.2) yields the following thermodynamic forces conjugate to the (elastic) strain *ϵ*, to *B*, and to the new family of state variables *s*_*i*_, respectively:

4.3a $$\begin{array}{rl} \sigma & =\frac{\partial \Psi }{\partial \epsilon }=f_{b}f_{s}\frac{\partial \psi_{r}}{\partial \epsilon }, \end{array}$$

4.3b $$\begin{array}{rl} E_{B} & =-\frac{\partial \Psi }{\partial B}=-\psi_{r}\frac{\partial f_{b}}{\partial B}f_{s} \end{array}$$

4.3c $$\begin{array}{rl} \text{and}\;\;\;\;E_{si} & =-\frac{\partial \Psi }{\partial s_{i}}=\psi_{r}f_{b}\frac{\partial f_{s}}{\partial s_{i}}\;\text{with}\;i=1,\ldots ,n. \end{array}$$

By retaining the validity of the breakage condition in equation (3.8), equation (4.3b) leads to the following expression of crushing pressure for linear and pressure-dependent elasticity:

4.4a $$p_{c}=p_{c}^{L}f_{s}^{1/2}f_{b}{\left(1-B\right)}^{-1}=p_{c}^{L}f_{s}^{1/2}\left(\frac{1-\theta B}{1-B}\right)$$

and

4.4b $$p_{c}=p_{c}^{N}f_{s}^{2/3}f_{b}{\left(1-B\right)}^{-(4/3)}=p_{c}^{N}f_{s}^{2/3}\frac{\left(1-\theta B\right)}{{\left(1-B\right)}^{4/3}},$$

in which the definitions of crushing pressure for assemblies made of spherical particles in equations (3.12) and (3.16) have been used. It is readily apparent that the incorporation of additional shape descriptors into the energy potential modifies the crushing resistance through multiplicative factors dependent on the selected type of elasticity and the value of the coefficient *f*_*s*_. Such effect will be examined in detail in the following sections with reference to specific examples.

The next key component for the extension of the CBM are coevolution laws for the shape descriptors. In agreement with the original CBM, here such laws are assumed to include a tendency towards a limit shape descriptor, *s*_*L*_. Furthermore, since in the context of confined comminution alterations of the particle shape are a direct consequence of particle ruptures, the shape descriptors are treated as passive variables, i.e. state variables whose evolution is a direct consequence of breakage (i.e. d*s*_*i*_ ∼ d*B*). Given the hypothesized coordinated evolution of *B* and the family of *s*_*i*_ descriptors, it is further postulated that at ultimate breakage all states variables converge to their respective attractor, which in mathematical terms implies:

4.5 dsi(1−B)2∼dB(sLi−si).

The equation above is boxed to highlight that the second key concept in this paper is the coevolution laws between the grain shape variable vector {*s*_1_, *s*_2_, …, *s*_*n*_} and grain size through the variable *B*. In particular, based on equation (4.5), the increment of a shape descriptor, d*s*_*i*_, scales with d*B* based on the relative distance between its current value and its attractor, $s_{L}^{i}$ (i.e. the further *s*_*i*_ is from $s_{L}^{i}$, the faster its evolution rate). In accordance with the breakage dissipation increment, equation (3.9b), the breakage rate scales with (1 − *B*)^2^, reflecting that *B* can only grow. By contrast, equation (4.5) implies that the shape descriptors can converge to their attractors either with increasing or decreasing trends, depending on whether their initial state is above or below $s_{L}^{i}$. The above considerations suggest the following expression for the coevolution laws of the family of *s*_*i*_ variables:

4.6 $$\text{d}s_{i}=\frac{c_{i}}{{\left(1-B\right)}^{2}}(s_{L}^{i}-s_{i})\;\text{d}B\;\text{with}\;i=1,\ldots ,n,$$

where, in addition to the introduction of dimensionless breakage-shape coevolution vector of constants *c*_*i*_, the rate of shape change is determined by the distance from the attractor $s_{L}^{i}$. Regarding this reference, it should be noted that the values of $s_{L}^{i}$ can be constrained with geometric arguments (figure 2), thus leaving the coevolution constants *c*_*i*_ as the only new parameters to be added to those of the original CBM framework for simulating particle shape evolution. The extended expression of Ψ and the evolution laws in equation (4.6) lead to a generalized form of the thermodynamic expressions presented previously, accordingly:

4.7 $$\text{d}\Phi =E_{B}\;\text{d}B+\sum_{i=1}^{n}E_{si}\;\text{d}s_{i}>0,$$

where *E*_*si*_ are thermodynamic forces work-conjugates to the newly defined shape descriptors. The formulation is completed by the following evolution equations:

4.8a $$\text{d}B=2\Lambda \frac{{\left(1-B\right)}^{2}}{E_{c}}$$

and

4.8b $$\text{d}s_{i}=2\Lambda \frac{c_{i}}{E_{c}}(s_{L}^{i}-s_{i}),$$

which are in agreement with the original CBM’s use of the breakage condition of equation (3.8). By considering equations (4.7) and (4.8), we will later identify restrictions to the admissible values of the family of constants *c*_*i*_, and in turn to the rate of shape evolution. In the following sections, such general formalism will be specialized to particular choices of shape descriptors by illustrating the use and performance of the proposed framework.

## 5. The coevolution of breakage and aspect ratio

### (a) Surface area and Helmholtz free energy

The incorporation of a specific dependence of Ψ on shape descriptors is based on the hypothesis of a linear scaling between the elastic strain energy and the surface area of the particles. The original version of this choice was motivated by DEM simulations with spheres [26], so its extension for non-spherical particles should be assessed and might be susceptible to future adjustments. A straightforward description of the surface area of arbitrarily shaped particles can be achieved by approximating them as ellipsoids, for which the shape of a grain is characterized by three principal dimensions, *L* > *I* > *S*, respectively. The surface area of an ellipsoid, $\Sigma_{A}$, can then be approximated with the Knud Thomsen formula:

5.1 $$\Sigma_{A}=4\pi {\left(\frac{L^{p}I^{p}+L^{p}S^{p}+I^{p}S^{p}}{3}\right)}^{1/p},$$

where *p* ≈ 1.6. Although the principal dimensions can be treated as independent variables, for simplicity this section refers to a prolate geometry (i.e. *I* = *S*). The case of oblate particles (i.e. *L* = *I*), not treated here, can in principle be examined by following a similar strategy. This restriction to the intermediate length enables tracking the shape of ellispoidal particles as follows:

5.2 $$\Sigma_{A}=4\pi {\left(\frac{2L^{p}S^{p}+S^{2p}}{3}\right)}^{1/p}=4\pi S^{2}{\left(\frac{1+2\alpha^{-p}}{3}\right)}^{1/p},$$

where the aspect ratio, *α*, is introduced according to the following definition:

5.3 $$\alpha =\frac{S}{L}\leq 1.$$

For simplicity, equation (5.2) can be linearized through Taylor expansion around *α* = 1, obtaining:

5.4 $$\Sigma_{A}=4\pi S^{2}\left(\frac{5-2\alpha }{3}\right).$$

The above suggests that the surface area, $\Sigma_{A}$: (i) scales with *S*^2^, implying that the smallest particle size plays a role similar to the grain size *x* in the original CBM; and (ii) accounts for the particle aspect ratio *α* through the coefficient

5.5 $$f_{s}\equiv f_{s}(\alpha )=\frac{5-2\alpha }{3},$$

whose effect vanishes at the limit of *α* = 1 (i.e. *f*_*s*_ = 1), when the longest and shortest dimensions coincide for a spherical geometry. It should be noted that, although the linearization in equation (5.4) has the benefit of leading to a linear dependence of *f*_*s*_ on the state variable *α* (similar to how *f*_*b*_ depends linearly on *B*), it loses accuracy for highly distorted particles (i.e. *α* ≈ 0). In such cases, the nonlinear dependence on *α* reflected in equation (5.2) can be used with no additional changes to the formulation. These results can be incorporated in an extended Helmholtz free energy potential, in that *f*_*s*_ represents the function *f*_*si*_ in equation (4.2). In agreement with equation (5.4), it therefore follows:

5.6 $$\Psi (\epsilon ,B,\alpha )=f_{b}f_{s}\psi_{r}=(1-\theta B)\left(\frac{5-2\alpha }{3}\right)\psi_{r}(\epsilon ).$$

### (b) Thermodynamic restrictions

Given equation (4.7), the dissipation increment can be expressed in terms of the increments of the two state variables, *B* and *α*:

5.7 $$\text{d}\Phi =E_{B}\;\text{d}B+E_{\alpha }\text{d}\alpha \geq 0,$$

which shows energy loss due to simultaneous size reduction and shape alteration. In the above, $E_{\alpha }$ is taken as the thermodynamic force associated with changes in aspect ratio, here referred to as *morphing energy*. Using the above equation and the energy balance in equation (3.6a), we find:

5.8 $$\left(\sigma -\frac{\partial \Phi }{\partial \epsilon }\right)\text{d}\epsilon +\left(E_{B}-\frac{\partial \Phi }{\partial B}\right)\text{d}B+\left(E_{\alpha }-\frac{\partial \Phi }{\partial \alpha }\right)\text{d}\alpha =0,$$

from which:

5.9a $$\begin{array}{rl} \sigma & \equiv \frac{\partial \Psi }{\partial \epsilon }=f_{b}f_{s}\frac{\partial \psi_{r}}{\partial \epsilon }, \end{array}$$

5.9b $$\begin{array}{rl} E_{B} & \equiv -\frac{\partial \Psi }{\partial B}=-\frac{\partial f_{b}}{\partial B}f_{s}\psi_{r} \end{array}$$

5.9c $$\begin{array}{rl} E_{\alpha } & \equiv -\frac{\partial \Psi }{\partial \alpha }=-f_{b}\frac{\partial f_{s}}{\partial \alpha }\psi_{r}. \end{array}$$

### (c) Stiffness

The expression of the Helmholtz free energy function in equation (5.6) enables the derivation of the elastic bulk modulus, *K*_*b*_, as follows:

5.10 $$K_{b}\equiv \frac{\partial^{2}\Psi }{\partial \epsilon^{2}}=f_{b}f_{s}\frac{\partial^{2}\psi_{r}}{\partial \epsilon^{2}}.$$

This expression can be specialized for the Helmholtz free energy functions introduced previously. In case of linear elasticity, its expression is given by:

5.11 $$K_{b}\equiv (1-\theta B)\left(\frac{5-2\alpha }{3}\right)K,$$

while for pressure-dependent elasticity:

5.12 $$K_{b}\equiv (1-\theta B)\left(\frac{5-2\alpha }{3}\right)p_{r}\bar{K}{\left[\bar{K}(1-m)\epsilon +1\right]}^{m/(1-m)}.$$

The latter can be further simplified by setting the constant $m=\frac{1}{2}$, as before, and expressing it in stress-dependent form through equation (5.9a). From these steps, it follows:

5.13 $$K_{b}\equiv \sqrt{1-\theta B}\sqrt{\frac{5-2\alpha }{3}}p_{r}\bar{K}\sqrt{\frac{\sigma }{p_{r}}}.$$

The above derivations highlight the consequences of the scaling hypotheses used for Ψ on the elastic properties of the continuum. Regardless of the specifics of the Helmholtz free energy functions, all the resulting expressions of elastic bulk modulus involve two scaling coefficients, one dependent on breakage, *B*, and another on the aspect ratio, *α*. Such coefficients incorporate the effect of evolving grain size polydispersity and particle shape, respectively.

It is interesting to note that both capture correctly the trend exhibited by experiments reported in the literature. Specifically, while the breakage-dependent term reflects the decrease in stiffness caused by a broadening of the GSD [52], the shape-dependent term determines an increase in bulk stiffness due to higher particle irregularity [20]. The latter result is thus consistent with findings by [21], in that, with all other state variables kept constant, it provides maximum stiffness away from the idealized spherical geometry (i.e. away from an aspect ratio *α* = 1 towards *α* = 0).

### (d) Yielding

The breakage energy in equation (5.9b) can be incorporated into the breakage criterion in equation (3.8) to infer yielding conditions. For linear elastic behaviour, it follows that the yielding stress takes the form:

5.14 $$p_{c}=p_{c}^{L}f_{s}^{1/2}f_{b}{\left(1-B\right)}^{-1}=p_{c}^{L}{\left(\frac{5-2\alpha }{3}\right)}^{1/2}\left(\frac{1-\theta B}{1-B}\right),$$

while for pressure-dependent elasticity it is given by

5.15 $$p_{c}=p_{c}^{N}f_{s}^{2/3}f_{b}{\left(1-B\right)}^{-(4/3)}=p_{c}^{N}{\left(\frac{5-2\alpha }{3}\right)}^{2/3}\frac{\left(1-\theta B\right)}{{\left(1-B\right)}^{4/3}}.$$

It is thus readily apparent that the theory predicts a shape-dependent corrective coefficient expressed as a function of the scaling function *f*_*s*_. Since CBM predicts a correlation between stiffness and yielding stress, the corrective coefficient mirrors the hypothesized influence of the particle shape on the bulk modulus (i.e. departure from spherical shapes implies an increase of surface area and a consequent stiffening of the material, with all other state variables kept constant, including the grain size through *B*). In other words, departure from sphericity is predicted to cause an increase in yielding stress. Although this result apparently contradicts some results available in the literature [23,24], it offers useful insight for a reexamination of the existing evidence. In fact, it points out that the origin of the often reported reduction of the yielding stress in assemblies made of irregular particles may not be directly caused by system effects affecting the elasticity of the particle packing, but rather by how the local grain irregularities modify the fracture mechanisms and the consequence energy threshold at the onset of comminution [48].

### (e) Coevolution law

Equation (5.6), along with the evolution equations of the inelastic state variables, enables the quantification of the dissipation increment and the consequent identification of the conditions satisfying the non-negativity restriction of the dissipation increment in equation (4.7). Specifically, in CBM the evolution of *B* is governed by an attractor associated with an ultimate GSD (*p*_*u*_ in equation (3.4)). A similar logic is used here for the state variable *α* by defining an ultimate value *α*_*L*_ serving as attractor. As pointed out by figure 2, this value is expected to lay within a narrow range (i.e. 0.43 < *α*_*L*_ < 0.63). By using the aforementioned attractor, it is possible to specialize the evolution equation (4.6), as follows:

5.16 $$\text{d}\alpha =\frac{c_{\alpha }}{{\left(1-B\right)}^{2}}(\alpha_{L}-\alpha )\;\text{d}B.$$

Integration of equation (5.16) from initially unbroken state (*B* = 0) leads to:

5.17 $$\alpha =\alpha_{L}+(\alpha_{0}-\alpha_{L})\;{\text{e}}^{-c_{\alpha }(B/(1-B))},$$

where *α*_0_ is the aspect ratio prior to breakage (i.e. at *B* = 0) and $c_{\alpha }$ is a constant controlling the rate of shape evolution in relation with the breakage growth rate.

Two scenarios will be considered here for such a coevolution constant: (i) $c_{\alpha }>1$, for which the shape evolves faster than the grain size reduction and meets its attractor before the system reaches the ultimate GSD (a case here referred to as *tachymorphic*); (ii) $c_{\alpha }<1$, for which the shape evolves at a slower rate than the size reduction and achieves its attractor after extensive comminution (a case here referred to as *brachymorphic*).

These scenarios have distinct thermodynamic implications. By inserting equation (5.16) and the breakage evolution equation (4.8a) into equation (5.7), it follows that the dissipation increment is:

5.18 $$\text{d}\Phi =2\Lambda \frac{{\left(1-B\right)}^{2}}{E_{c}}E_{B}\left(1+\frac{2c_{\alpha }}{\theta }\frac{1-\theta B}{{\left(1-B\right)}^{2}}\frac{\alpha_{L}-\alpha }{5-2\alpha }\right)\geq 0,$$

which can be used to set restrictions to the coevolution constant $c_{\alpha }$. To have non-negative dissipation, $c_{\alpha }$ has to be within a specific range, $c_{\alpha }^{\text{min}}<c_{\alpha }<c_{\alpha }^{\text{max}}$. The upper limit for $c_{\alpha }$ can be understood by examining the effect of shape alterations on the energy balance. For granular media with highly distorted particles (i.e. 0 < *α* < *α*_*L*_) positive dissipation is always guaranteed, regardless the value of the evolution coefficient $c_{\alpha }$. This is a consequence of elongated particles gradually taking a more spherical shape, by losing elongation features which provide further contact sites for energy storage. By contrast, relatively undistorted grains (i.e. *α*_*L*_ < *α* < 1) lead to negative contributions to the dissipation rate, as they start with relatively spheroidal shapes which are progressively distorted by crushing. In this scenario grain ruptures create elongated features, which may offer more frequent opportunities for contact formation, thus counteracting the energy loss due to size reduction (i.e. loss of suitable sites for force chain development). The most restrictive value for $c_{\alpha }^{\text{max}}$ can then be obtained by referring to the highest admissible value of *α*=1, for which the negative contributions to equation (5.18) are maximized. Such effects are stronger at the start of the breakage process (i.e. *B* = 0) and result in $c_{\alpha }^{\text{max}}=3\theta /[2(1-\alpha_{L})]$. While this restriction provides a cap for the rate of tachymorphism, it needs to be complemented by a lower limit of $c_{\alpha }$. For the reasons mentioned above, the latter is relevant only for nearly spherical initial shapes. However, in case of brachymorphism the shape is altered weakly during comminution, thus experiencing sharp variation only at high values of *B*. As a result, the assessment of $c_{\alpha }^{\text{min}}$ is more complex, as it requires consideration of the coordinated evolution of *B* and *α*. Details about the determination of $c_{\alpha }^{\text{min}}$ are provided in appendix A. However, it is possible to show that a conservative choice for the lower limit is $c_{\alpha }^{\text{min}}=1/20$, which can be used for a first-order estimate. For all practical purposes, the above formulation satisfies the non-negativity of the dissipation rate by adopting:

5.19 $$\frac{1}{20}\leq c_{\alpha }\leq \frac{3\theta }{2(1-\alpha_{L})}.$$

### (f) Simulations

**Figure 4.**
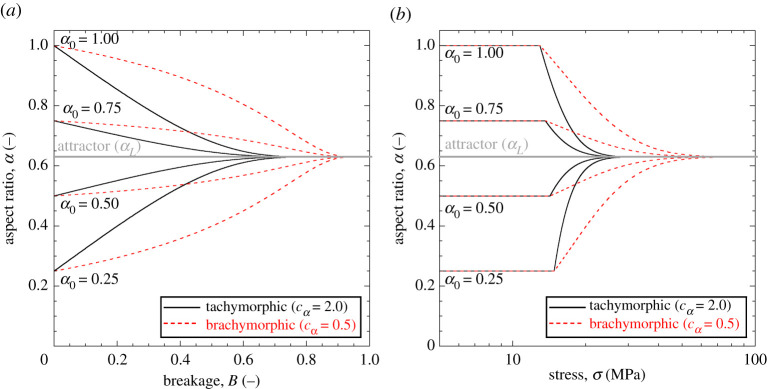
Evolution of the aspect ratio of the grains, *α*, as a function of breakage, *B* (*a*), and stress, *σ* (*b*), with reference to different initial values of *α* prior to breakage. Simulations based on $\bar{K}=8$, *m* = 0.5, *p*_*r*_ = 1 MPa, *E*_*c*_ = 3.5 MPa, *θ* = 0.9, and $\alpha_{L}=1/\sqrt[{3}]{2^{2}}$. Tachymorphic examples based on $c_{\alpha }=2.0$. Brachymorphic examples based on $c_{\alpha }=0.5$. (Online version in colour.)

Given the simplicity of the above formulation all the analyses discussed in this section are based on a pressure-dependent elastic model introduced in §3. Figure 4 illustrates simulations conducted for different initial values of aspect ratio. Convergence to the attractor *α*_*L*_ is shown as a function of breakage and stress, with the value of $c_{\alpha }$ influencing the trend of convergence. Tachymorphic behaviour ($c_{\alpha }>1$) implies sharp approach of the attractor right after yielding. In this case, the model predicts a steady evolution of the shape descriptor well before the ultimate GSD (i.e. the particles continue to decrease in size, but the new fragments possess a similar aspect ratio to that of their progenitors). Such a scenario leads to sharp shape-stress evolution curves (figure 4*b*), with marked changes in yielding stress and, consequently, strain increments. By contrast, brachymorphic behaviour ($c_{\alpha }<1$) leads to slower convergence to the attractor. This is reflected by negligible shape changes in the early stages of breakage (figure 4*a*) and weaker variation of the particle shape upon loading (figure 4*b*). In this scenario, the model predicts that the attractor is reached close to the ultimate GSD, thus requiring large stress to reach its final value.

**Figure 5.**
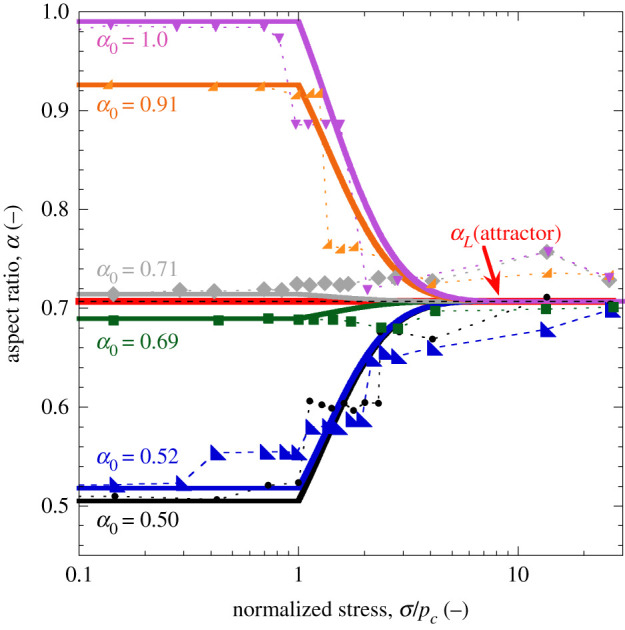
Continuum model performance in replicating the evolution of the aspect ratio of grains resulting from two-dimensional DEM simulations by Ueda *et al*. [43]. Analysis based on $\bar{K}=8$, *m* = 0.5, *p*_*r*_ = 1 MPa, *E*_*c*_ = 3.5 MPa, *θ* = 0.9, $\alpha_{L}=1/\sqrt{2}=2^{-(1/2)}$ and $c_{\alpha }=0.4$. Results reported in term of normalized stress (*p*_*c*_ refers to the stress at the onset of crushing). (Online version in colour.)

The model was tested against results available in the literature. Figure 5 shows a comparison between CBM simulations and DEM analyses reported by [43] for two-dimensional crushable agglomerates with ellipsoidal and polygonal shapes. The calibration of the CBM model parameters focused on the coevolution constant $c_{\alpha }$, in that all other material constants were set to values consistent with previous calibrations for crushable granular materials [25,53]. Although the discrete nature of the DEM analyses inevitably involves sharp jumps in aspect ratio, the results reported by Ueda *et al*. [43] display asymptotic values oscillating within a narrow range. Use of a unique ultimate aspect ratio, *α*_*L*_ in the CBM analyses (i.e. $\alpha_{L}=1/\sqrt{2}$, corresponding to the two-dimensional silver ratio [42]) therefore leads to a satisfactory match between the previously published discrete and current continuum analyses.

**Figure 6.**
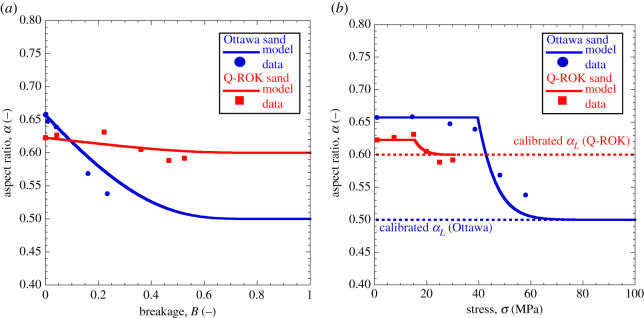
Model performance in replicating the evolution of the aspect ratio of sands subjected to confined compression: (*a*) Ottawa sand; (*b*) Q-ROK sand. Simulations of Ottawa sand based on $\bar{K}=15$, *m* = 0.5, *p*_*r*_ = 1 MPa, *E*_*c*_ = 9.0 MPa, *θ* = 0.9, *α*_*L*_ = 0.5 and $c_{\alpha }=2.7$. Simulations of Q-ROK sand based on $\bar{K}=12$, *m* = 0.5, *p*_*r*_ = 1 MPa, *E*_*c*_ = 2.7 MPa, *θ* = 0.9, *α*_*L*_ = 0.6 and $c_{\alpha }=2.0$. Data after [28]. (Online version in colour.)

Figure 6 illustrates a similar example with reference to shape evolution data reported by Seo *et al*. [28]. In this case, two quartz sands with subrounded (Ottawa) and subangular (Q-ROK) particles, respectively, were crushed through one-dimensional compression and scanned using X-ray tomography. While the applied stress was not sufficient to clearly identify an asymptotic trend, the figure shows that the measurements can be accurately replicated by the proposed CBM formulation. In this case, the calibration of the model parameters involved both the coevolution constant $c_{\alpha }$ and the attractor *α*_*L*_. While the former was constrained based on measurements of coevolution of breakage and aspect ratio (figure 6*a*), the latter was optimized to capture changes of particle shape upon loading (figure 6*b*). The simulations display a satisfactory match of the shape evolution measurements in terms of both breakage and pressure. Despite the similarity of the initial aspect ratio of the two sands, their ultimate values of *α*_*L*_ were found different (*α*_*L*_ = 1/2 for Ottawa and *α*_*L*_ = 3/5 for Q-ROK sand). Such variations may be regarded as a product of the different formation history of the two sands, with Ottawa being the outcome of fluvial deposition, and Q-ROK deriving from artificial grinding. Regardless of these differences, in both cases the estimated shape attractors were consistent with evidence from extreme fragmentation in nature (figure 1). In addition, the measured trends could be captured only by using values of $c_{\alpha }>1$ (tachymorphic behaviour), according to which the achievement of the ultimate GSD (*B* = 1) occurs when the shape attractor has nearly been achieved (i.e. size reduction is predicted to continue to occur while the aspect ratio of the particles remains practically constant).

## 6. The coevolution of breakage, flatness and elongation

### (a) Surface area and Helmholtz free energy

**Figure 7.**
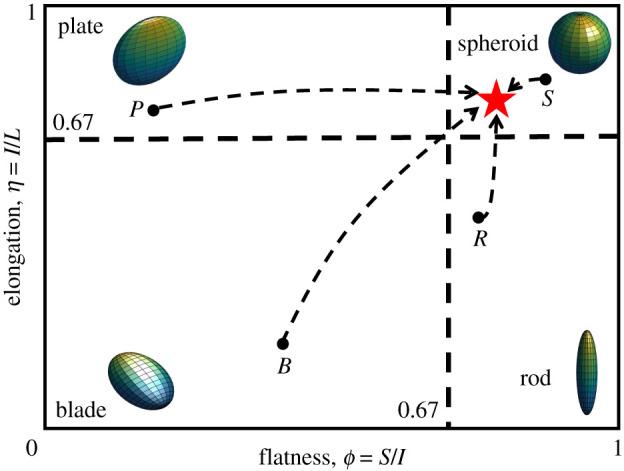
Representation of particle shape evolution in the morphometric plane. Four initial states are depicted, each associated with a distinct class of particle shape: spheroid (*S*), plate (*P*), rod (*R*) and blade (*B*). Convergence towards a shape attractor (star symbol) is also represented. (Online version in colour.)

To express the surface area as a function of multiple shape descriptors, we use the binary set of variables proposed by Zingg [54]. Similar to the aspect ratio, here too deviations from the ideal case of a sphere are encapsulated into non-dimensional ratios between principal lengths. In this case, the two selected state variables are flatness and elongation, being *ϕ* = *S*/*I* and *η* = *I*/*L*, respectively. A visual interpretation of the particle geometry associated with these parameters is provided in figure 7. Low flatness ratio *ϕ* corresponds with a shortest dimension much smaller than the others, thus constituting the thickness of a flat object (upper and lower left quadrants in figure 7, corresponding to shape classes defined as *plates* and *blades*). Similarly, low elongation ratio *η* denotes shapes in which the maximum dimension is dominant over the intermediate length, such as in rod-shaped particles (lower right quadrant). Comparable, high values of the two ratios correspond to spheroidal particles (upper right quadrant). The selection of *ϕ* and *η* as state variables enables to use the Zingg diagram, hereafter referred to as *morphometric plane*, to track the evolution of the particle shape during breakage. This is depicted in figure 7, where the convergence of different initial states to a prescribed attractor is depicted.

Different choices of initial *ϕ* and *η* lead to shapes that range from oblate (rod-like) to highly prolate (plate-like), thus greatly influencing the value of particle surface area, $\Sigma_{A}$. Such effects can be captured by the surface area expression in equation (5.1), which can be expressed as a function of the chosen state variables as follows:

6.1 $$\Sigma_{A}=4\pi I^{2}\sqrt[{p}]{\frac{\eta^{-p}+\eta^{-p}\phi^{p}+\phi^{p}}{3}}\approx 4\pi I^{2}\left(1+\frac{2}{3}(\phi -\eta )\right),$$

where linearization around *ϕ* = 1 and *η* = 1 has been performed.

This treatment implies that $\Sigma_{A}$ scales with both: (i) *I*^2^, meaning that the intermediate length plays the role of the grain size *x* of the original CBM; and linearly with (ii) the following shape-dependent coefficient

6.2 $$f_{s}\equiv f_{s}(\phi ,\eta )=1+\frac{2}{3}(\phi -\eta ).$$

The influence of *f*_*s*_ vanishes at the limit *ϕ* ≡ *η* ≡ 1 (i.e. *f*_*s*_ = 1 when all the principal dimensions coincide, as in spherical grains). Such a result can be incorporated into an extended Helmholtz free energy potential, as follows:

6.3 $$\Psi (\epsilon ,B,\phi ,\eta )=(1-\theta B)(1+\frac{2}{3}(\phi -\eta ))\psi_{r}(\epsilon ).$$

### (b) Thermodynamic restrictions

The dissipation increment can then be expressed by:

6.4 $$\text{d}\Phi =E_{B}\;\text{d}B+E_{\phi }\;\text{d}\phi +E_{\eta }\;\text{d}\eta \geq 0,$$

where $E_{\phi }$ and $E_{\eta }$ are the thermodynamic forces associated with the particle elongation and flatness, and are thus defined as the *elongation energy* and *flatness energy*, respectively. From equation (6.4), it follows that:

6.5 $$\left(\sigma -\frac{\partial \Phi }{\partial \epsilon }\right)\text{d}\epsilon +\left(E_{B}-\frac{\partial \Phi }{\partial B}\right)\text{d}B+\left(E_{\phi }-\frac{\partial \Phi }{\partial \phi }\right)\text{d}\phi +\left(E_{\eta }-\frac{\partial \Phi }{\partial \eta }\right)\text{d}\eta =0,$$

from which

6.6a $$\begin{array}{rl} \sigma & =\frac{\partial \Psi }{\partial \epsilon }=(1-\theta B)\left(1+\frac{2}{3}(\phi -\eta )\right)\frac{\partial \psi_{r}}{\partial \epsilon }, \end{array}$$

6.6b $$\begin{array}{rl} E_{B} & =-\frac{\partial \Psi }{\partial B}=\theta \left(1+\frac{2}{3}(\phi -\eta )\right)\psi_{r}(\epsilon ), \end{array}$$

6.6c $$\begin{array}{rl} E_{\phi } & =-\frac{\partial \Psi }{\partial \phi }=-\frac{2}{3}(1-\theta B)\psi_{r} \end{array}$$

6.6d $$\begin{array}{rl} \text{and}\;\;\;\;E_{\eta } & =-\frac{\partial \Psi }{\partial \eta }=\frac{2}{3}(1-\theta B)\psi_{r}. \end{array}$$

### (c) Stiffness

The expression of the Helmholtz free energy function in equation (6.3) enables the derivation of the elastic bulk modulus, as follows:

6.7 $$K_{b}\equiv \frac{\partial^{2}\Psi }{\partial \epsilon^{2}}=(1-\theta B)\left(1+\frac{2}{3}(\phi -\eta )\right)\frac{\partial^{2}\psi_{r}}{\partial \epsilon^{2}}.$$

This expression can be specialized for the Helmholtz free energy functions introduced previously. In the case of linear elasticity, its expression is given by:

6.8 $$K_{b}\equiv (1-\theta B)(1+\frac{2}{3}(\phi -\eta ))K,$$

while for pressure-dependent elasticity:

6.9 $$K_{b}\equiv (1-\theta B)(1+\frac{2}{3}(\phi -\eta ))p_{r}\bar{K}{\left[\bar{K}(1-m)\epsilon +1\right]}^{m/(1-m)}.$$

The latter can be simplified by adopting $m=\frac{1}{2}$ and expressing the bulk modulus in a stress-dependent form:

6.10 $$K_{b}\equiv \sqrt{1-\theta B}\sqrt{1+\frac{2}{3}(\phi -\eta )}p_{r}\bar{K}\sqrt{\frac{\sigma }{p_{r}}}.$$

Once again, these results provide scaling trends consistent with available evidence about the role of grain size and shape on the elastic properties of sands [20,21,52]. Specifically, the expressions above also predict an increase in bulk stiffness with particle irregularity, which is reflected by the approach of minimum stiffness values corresponding to the case of spherical particles (i.e. when *η* = *ϕ* = 1).

### (d) Yielding

The breakage energy in equation (6.6b) can be incorporated into equation (3.8) to infer the yielding threshold. For a linear elastic behaviour, it follows that:

6.11 $$p_{c}=p_{c}^{L}{\left(1+\frac{2}{3}(\phi -\eta )\right)}^{1/2}\left(\frac{1-\theta B}{1-B}\right)=p_{c}^{L}f_{s}^{1/2}\left(\frac{1-\theta B}{1-B}\right),$$

while for pressure-dependent elasticity:

6.12 $$p_{c}=p_{c}^{N}{\left(1+\frac{2}{3}(\phi -\eta )\right)}^{2/3}\frac{\left(1-\theta B\right)}{{\left(1-B\right)}^{4/3}}=p_{c}^{N}f_{s}^{2/3}\frac{\left(1-\theta B\right)}{{\left(1-B\right)}^{4/3}}.$$

Again, we find that departure from sphericity leads to an increase in yielding resistance, and that the evolution of grain shape adjusts the progress of clastic hardening.

### (e) Coevolution laws

The quantification of the dissipation increment for this generalized model requires coevolution equations for the inelastic grain size (through *B*) and shape state variables (through *ϕ* and *η*). In analogy with the aspect ratio formulation in the previous section, here these are given by

6.13a $$\text{d}\phi =\frac{c_{\phi }}{{\left(1-B\right)}^{2}}(\phi_{L}-\phi )\;\text{d}B$$

and

6.13b $$\text{d}\eta =\frac{c_{\eta }}{{\left(1-B\right)}^{2}}(\eta_{L}-\eta )\;\text{d}B.$$

Integration from an initially unbroken state (*B*=0) leads to:

6.14a $$\phi =\phi_{L}+(\phi_{0}-\phi_{L})\;{\text{e}}^{-c_{\phi }(B/(1-B))}$$

and

6.14b $$\eta =\eta_{L}+(\eta_{0}-\eta_{L})\;{\text{e}}^{-c_{\eta }(B/(1-B))},$$

where *ϕ*_0_ and *η*_0_ are the initial flatness and elongation ratios, while $c_{\phi }$ and $c_{\eta }$ are constants controlling the rate of shape evolution. The coevolution laws hypothesize convergence of the shape descriptors to their attractors (*ϕ*_*L*_ and *η*_*L*_, respectively). Ranges for the attractor point can be set on the basis of figure 2, which suggests 0.65 < *ϕ*_*L*_ ≈ *η*_*L*_ < 0.8. While this formalism does not directly track the aspect ratio, its value can be readily computed as *α* = *ϕη*. Similarly, the sphericity, *ψ*_*S*_, can also be tracked through the Krumbein approximation [55], according to which $\psi_{S}=\sqrt[{3}]{IS/L^{2}}=\sqrt[{3}]{\phi \eta^{2}}$. These relations simply expand the possibilities for depicting the evolution of various shape parameters.

While equation (6.14) enables both tachymorphic ($c_{\phi }>1$ and $c_{\eta }>1$) and bracymorphic behaviours ($c_{\phi }<1$ and $c_{\eta }<1$), the coexistence of two attractors leads to more demanding thermodynamic restrictions for the value of the coevolution constants. Such restrictions can be defined by specializing the dissipation increment for the case of the evolution laws in equation (6.13):

6.15 $$\text{d}\Phi =2\Lambda \frac{{\left(1-B\right)}^{2}}{E_{c}}E_{B}\left(1+\frac{2}{3\theta }\frac{1-\theta B}{{\left(1-B\right)}^{2}}\frac{c_{\phi }(\phi -\phi_{L})-c_{\eta }(\eta -\eta_{L})}{1+\frac{2}{3}(\phi -\eta )}\right)\geq 0.$$

**Figure 8.**
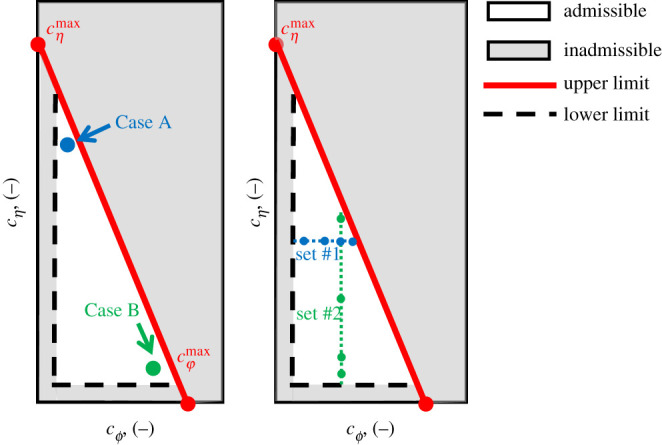
Admissible range of coevolution parameters. The diagram on the left illustrates the thermodynamic restrictions for the coevolution constants $c_{\phi }$ and $c_{\eta }$ $\left((\mathrm{with})c_{\phi }^{\text{max}}=\frac{2}{\theta \phi_{L}}c_{\eta }^{\text{max}}=\frac{2}{\theta (\eta_{L}-1)}\right)$). It also depicts two example combinations close to the limits of admissibility, labelled as Case A ($c_{\eta }=1.50$; $c_{\phi }=0.075$) and Case B ($c_{\eta }=0.075$; $c_{\phi }=0.50$). By contrast, the diagram on the right depicts different sets of combinations for $c_{\eta }$ and $c_{\phi }$. Set #1 involves $c_{\eta }=0.75$ and $c_{\phi }=\{0.075,0.15,0.25\text{ or }0.35\}$. Set #2 involves $c_{\phi }=0.25$ and $c_{\eta }=\{0.075,0.15,0.45\text{ or }0.90\}$. (Online version in colour.)

Fulfilment of the sign restriction in equation (6.15) implies an admissible range for the evolution coefficients (i.e. $c_{j}^{\text{min}}<c_{j}<c_{j}^{\text{max}}$, with *c*_*j*_ representing either $c_{\phi }$ or $c_{\eta }$). The upper limits for *c*_*j*_ can be defined by examining equation (6.15) for the most restrictive state, i.e. when *ϕ* = 0 and *η* = 1 (i.e. plate-like particles, for which the negative contributions into equation (6.15) are maximized). At this limit, $c_{\phi }$ and $c_{\eta }$ have to satisfy the following relation:

6.16 $$1+\frac{2}{\theta }(c_{\phi }(\phi -\phi_{L})-c_{\eta }(\eta -\eta_{L}))\geq 0,$$

which can be described by the admissibility diagram in figure 8 for the two evolution coefficients. The diagram shows that the maximum rate of evolution for the elongation ratio *η* can be enforced when the shape changes with constant flatness ($c_{\phi }=0$ and d*ϕ* = 0). Such a limit value corresponds to $c_{\eta }^{\text{max}}=2/(\theta (\eta_{L}-1))$. Conversely, lack of evolution for *η* (i.e. $c_{\eta }=0$ and d*η* = 0) enables the maximum rate of variation for the flatness ratio *ϕ*, associated with $c_{\phi }^{\text{max}}=2/(\theta \phi_{L})$. In general these restrictions are more severe than those previously discussed for the aspect ratio model and imply that tachymorphic behaviour is not always allowed. Specifically, tachymorphic behaviour can be enforced only for the elongation (i.e. an admissible range of $c_{\eta }>1$ exists), while values of $c_{\phi }>1$ are not admissible for typical ranges of the parameters. In addition to the restriction to the maximum rate of shape evolution, restrictions for the lowest admissible values of the coefficients *c*_*j*_ are also required. The assessment of the values of $c_{j}^{\text{min}}$ is similar to the case of a single shape descriptor and a simplified procedure to define it is discussed in appendix A. Such a procedure leads to the same minimum value for both the coevolution coefficients, which, although derived for the restrictive case of $c_{\phi }=c_{\eta }$, can be used to set conservative limitations across the entire diagram in figure 8. This procedure leads to an L-shaped band at the bottom left corner of the plane, which in conjunction with the upper limit for the coevolution constant defines the inadmissible zone for combinations of $c_{\phi }$ and $c_{\eta }$ (grey-shaded zone). The remaining portion of the domain in figure 8 defines instead the admissible combinations of $c_{\phi }$ and $c_{\eta }$ (white zone). These restrictions to the coevolution parameters imply that this generalized formulation can only be employed in materials displaying a relatively slow evolution of the particle morphology. Not all materials, however, may conform to this trend (see for instance figure 6*a*, where X-ray data indicate tachymorphic patterns). This result can be interpreted as an outcome of the simplified structure of the coevolution laws in equation (6.13) and suggests that more elaborate expressions may be needed to accommodate experimental data. Nevertheless, this simple generalized model can offer insight on the capabilities of the proposed formulation, which will be explored hereafter.

### (f) Simulations

**Figure 9.**
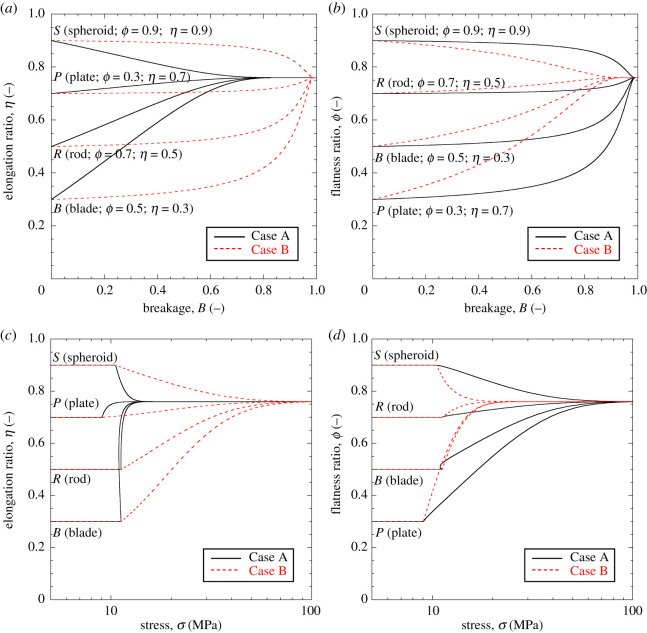
Evolution of flatness, *ϕ*, and elongation, *η*, as a function of breakage, *B*, (*a*, *b*) and stress, *σ*, (*c*, *d*). Simulations performed with reference to the four representative initial states in figure 7. Results obtained with parameters *K* = 10 MPa, *E*_*c*_ = 5.0 MPa, *θ* = 0.9, and *η*_*L*_ = *ϕ*_*L*_ = 0.76. Coevolution constants from two combinations depicted in figure 8 (i.e. Case A and Case B). (Online version in colour.)

This section uses CBM simulations to illustrate the key features of a model with multiple shape descriptors. Given the simplified nature of the analyses, the simulations have been conducted through a linear elastic strain energy model. Figure 9 illustrates the evolution of the elongation and flatness ratios as a function of breakage (figure 9*a*,*b*) and pressure (figure 9*c*,*d*). The analysis is run for two combinations of $c_{\phi }$ and $c_{\eta }$ values, referred to as Case A and Case B, respectively (figure 8). Case A reflects a scenario in which the rate of elongation evolution is maximized within the admissible range, while Case B represents the opposite scenario (i.e. near-maximum rate of flatness evolution). In all cases the shape descriptors converge towards the attractor at ultimate breakage and high stress (in this case, *ϕ*_*L*_ = *η*_*L*_ = 0.79). However, while Case B involves brachymorphic evolution for both shape descriptors (i.e. achievement of limit values of *ϕ* and *η* at high breakage, *B*), Case A implies tachymorphic evolution for elongation and brachymorphic trends for flatness. In other words, in Case A elongation evolves in a self-similar manner during breakage (i.e. fragments possess essentially the same elongation ratio as their progenitors for much of the loading process), while flatness changes weakly until high pressures. By contrast, in Case B high breakage is needed to obtain appreciable shape changes for both descriptors. In all cases, the value of the coefficients *c*_*j*_ is inversely related to the stress at which the attractor is approached, with higher values of *c*_*j*_ leading to relatively low stress at the achievement of the shape attractor.

**Figure 10.**
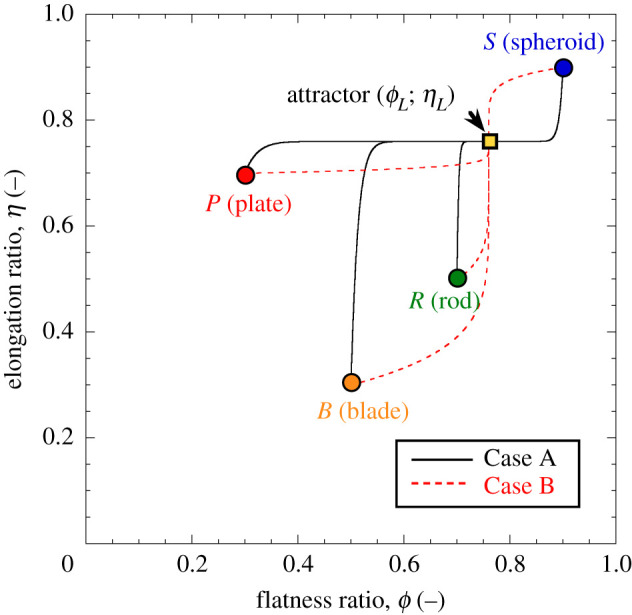
Shape evolution paths relative to the simulations in figure 9 depicted in the morphometric plane. (Online version in colour.)

The simulation results can also be visualized in the morphometric plane (figure 10). The use of combinations of coefficients *c*_*j*_ close to the limits of admissibility for $c_{\phi }$ and $c_{\eta }$ causes nonlinear shape evolution paths. Most notably, Case A corresponds to quasi-vertical initial paths (i.e. the elongation changes at near-constant flatness upon initial breakage), eventually switching to pseudo-horizontal paths when the elongation attractor is reached. By contrast, Case B initially involves quasi-horizontal paths (i.e. change of flatness at near-constant elongation), which eventually convert into vertical paths until reaching the attractor point. Such trends are found for all the considered initial shapes.

**Figure 11.**
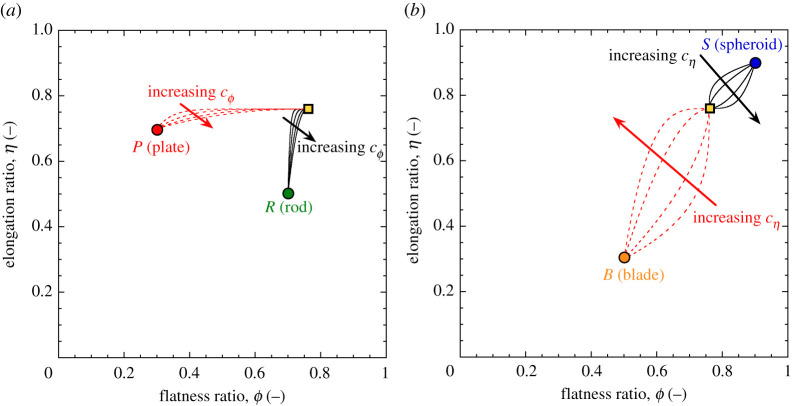
Shape evolution paths relative to the two sets of values of the coevolution constants $c_{\phi }$ and $c_{\eta }$ depicted in figure 8 (i.e. (*a*) Set #1 and (*b*) Set #2). (Online version in colour.)

To further illustrate the broad range of paths covered with suitable choices of the evolution coefficients, a parametric analysis has been conducted with reference to intermediate values of $c_{\phi }$ and $c_{\eta }$. Namely, the combinations included in Set #1 (figure 8) involve constant $c_{\eta }=0.75$ and increasing $c_{\phi }$. Similarly, the combinations included in Set #2 involve constant $c_{\phi }=0.25$ and increasing $c_{\eta }$. The resulting simulations are shown in figure 11 with reference to Set #1 (figure 11*a*, for initial shapes belonging to the classes of plates and rods) and Set #2 (figure 11*b*, for initial shapes belonging to the classes of plates and rods).

**Figure 12.**
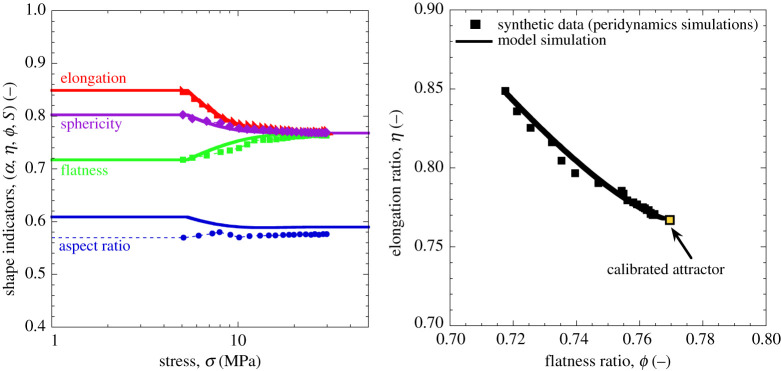
Comparison between simulations of particle shape evolution. Symbols refer to grain-scale fracture simulations conducted through a peridynamics engine (after [56,57]). Solid lines refer to continuum simulations based on the extended version of CBM. Results obtained with parameters *K* = 10 MPa, *E*_*c*_ = 1.4 MPa, *θ* = 0.9, and *η*_*L*_ = *ϕ*_*L*_ = 0.77, $c_{\phi }=0.15$ and $c_{\eta }=0.20$. (Online version in colour.)

Similar simulations are illustrated in figure 12 with reference to a multi-scale numerical engine allowing grain-scale fracture analyses through a peridynamics solver [56,58]. The results show that with a suitable choice of the attractor point it is possible to replicate satisfactorily the evolution of a number of morphological indicators. In this case, the calibrated attractor matching the grain-scale fracture simulations is *ϕ*_*L*_ = *η*_*L*_ = 0.768, which is well within the range of potential values in figure 2. Interestingly, the analysis indicates that the tracking of a single shape descriptor may be insufficient to identify significant geometric alterations. This is clearly shown in figure 12 by the weak evolution of the aspect ratio *α* resulting from more intense (but opposite) changes of the flatness, *ϕ*, and elongation, *η*, ratios.

**Figure 13.**
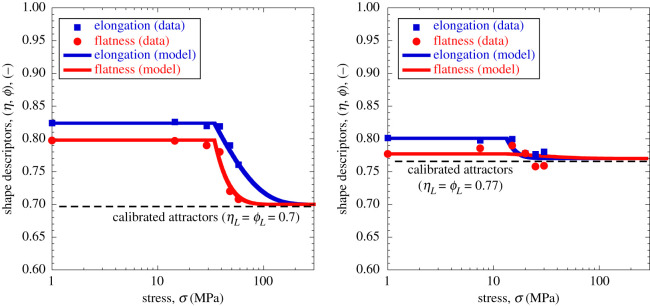
Comparison between measurements of particle shape evolution and CBM simulations. Symbols refer to X-ray tomography data (after [28]). Solid lines refer to continuum-scale simulations. Simulations of Ottawa sand based on *K* = 60 MPa, *E*_*c*_ = 9.0 MPa, *θ* = 0.9, and *η*_*L*_ = *ϕ*_*L*_ = 0.70, $c_{\phi }=0.30$ and $c_{\eta }=0.10$. Simulations of Q-ROK sand based on K=30 MPa, *E*_*c*_ = 2.7 MPa, *θ* = 0.9, and $\eta_{L}=\phi_{L}=1/\sqrt[{3}]{2}$, $c_{\phi }=0.05$ and $c_{\eta }=0.50$. (Online version in colour.)

**Figure 14.**
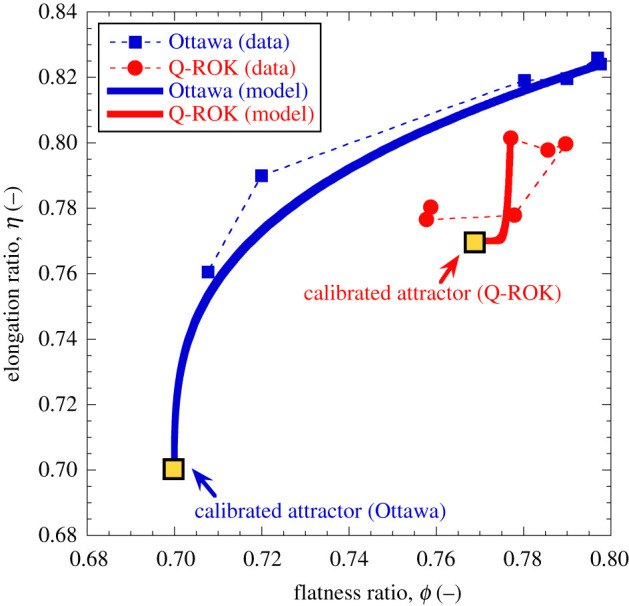
Comparison between paths of particle shape evolution depicted in the morphometric plane. Symbols refer to X-ray tomography data (after [28]). Solid lines refer to continuum-scale simulations based on the extended CBM formulation. (Online version in colour.)

Figures 13 and 14 finally compare the measurements previously discussed on Ottawa and Q-ROK sands [28] with the newly proposed CBM framework with the two shape descriptors. Values of shape attractors consistent with those used in the single-variable model were used. This choice leads to a satisfactory agreement between the constitutive model simulations and the experimental measurements, with rounded Ottawa sand being again characterized by stronger shape alterations compared with the more angular Q-ROK sand. The shape evolution paths are reproduced with reasonable accuracy in case of Ottawa sand, which exhibits stronger, yet smoother evolution trends. The first-order features of the shape evolution paths of Q-ROK sand are also captured reasonably well, albeit the measurement fluctuations of the average shape in the proximity of the attractor point, potentially due to the inherent difficulty of capturing small experimental variations in this particular regime.

## 7. Conclusion

By including grain shape descriptors other than the grain size into CBM, this paper offers a platform to inspect the implications of one of the most common, yet seldom tested, simplifications of granular mechanics models: the representation of particles as spherical bodies. The proposed theory expands the current scope of CBM by incorporating the effects of particle shape into macroscopic elasticity, yielding, and post-yielding compressibility. In addition, the generalized CBM theory also predicts the possibility of two end-members for the coevolution of particle size and shape—here defined as *tachymorphic* and *brachymorphic*—on the basis of the relative rates of change of the morphological descriptors. While these results involved several assumptions, they opened new research questions that should assist future studies about the feedback between particle shape and macroscopic deformation processes in granular media. Among such questions, an open challenge remains the rigorous identification of how macroscopic properties scale with grain shape descriptors. In our work, such connection emerges naturally from the hypothesized linear scaling between a particle’s surface area and bulk elastic stiffness. Our findings show that this choice has wide implications, as it influences both pre-yielding reversible processes as well as inelastic yielding stress and ensuing hardening/softening mechanisms. Future testing, validation and refinement of this hypothesis can therefore be conducted through grain-scale micromechanical analyses, focusing on how the shape of the particles affects global and collective elastic strain energy storage and mechanical dissipation [18,48]. This opens abundant opportunities for integrating the continuum-scale characterization proposed in this paper with the rising wave of computational methods for arbitrarily shaped particles, including DEM techniques relying on high-fidelity replicas of sand grains [14,59].

Another fundamental building block resulting from this paper is the use of coevolution laws dependent on ultimate attractors for grain size and shape. This idea proved instrumental to capture a diverse set of evidence, including DEM results, grain-scale fracture simulations and measurements of confined comminution based on X-ray tomography. The use of an attractor for grain shape was coordinated with the attraction to an ultimate fractal GSD in a fully crushed granular system. Here, the underlying working hypothesis is that both ultimate states are achieved at the end of comminution, with obvious implications for the energetics of the process and the consequent thermodynamic restrictions of the model parameters. Given the scarcity of available data about this topic, there are obvious opportunities for experimental, theoretical and computational research to clarify if these attractors depend on the past history of crushing, which grain-scale mechanisms control the rates of the coevolution, and, most importantly, whether the evolution of polydispersity may halt comminution and prevent ultimate shape attractors from being realized independently. At this juncture, an open area of research not explored in this work is how the inevitable statistical disorder of the particle shapes within granular materials influences the link between grain-scale and continuum-scale properties. From this standpoint, considerable assistance may derive from the combination of the proposed continuum framework with multi-scale characterization technologies based on digital imaging and high-resolution X-ray tomography, through which particle irregularities can be detected down to sub-micrometre scales, including alterations caused by grain crushing [8,60].

Finally, our findings regarding the connections among particle shape, energetics of grain-scale fracture and macroscopic comminution offer exciting opportunities to develop novel physics-based methods for granular material design [61–63]. In this context, a challenge that has not yet been fully explored is the non-isotropic connotation of particle shapes, in that any departure from a spherical geometry involves the incorporation of directional properties, which can in turn impact all aspects of the macroscale response. Recent work in this context offers guidance on how to describe the granular fabric in light of shape descriptors [64], as well as to incorporate it into CBM frameworks [65]. The unification of these research ideas should therefore provide a promising avenue for material optimization, design and manufacturing.

## Data accessibility

All data needed to evaluate the conclusions of this work are included in the paper or in the cited references.

## Appendix A

The maximum values for the coevolution constants controlling the rate of particle shape evolution can be defined in a relatively straightforward manner by setting the most restrictive conditions for the initial value of the corresponding shape descriptors. The strategy to compute it resulted in values of $c_{\alpha }^{\text{max}}$ (aspect ratio model), $c_{\phi }^{\text{max}}$ and $c_{\eta }^{\text{max}}$ (flatness-elongation model) expressed in analytical form as a function of the corresponding shape attractors (*α*_*L*_, *ϕ*_*L*_ and *η*_*L*_, respectively) and of the grading index, *θ*.

By contrast, the identification of the minimum value of these coevolution constants is not equally straightforward, in that it tends to be constrained by states located in the proximity of the ultimate breakage condition (i.e. *B* = 1), thus requiring consideration of both breakage and particle shape evolution. While a closed form expression for the lower limits of $c_{\alpha }$, $c_{\phi }$ and $c_{\eta }$ is not available, here an approximate strategy to constrain the lower bound of their admissible range is provided.

The strategy can be readily illustrated for the single-variable formulation based on the aspect ratio *α*. Minimization of the dissipation increment in equation (5.18) provides the breakage state, $\bar{B}$, at which the lowest value of dissipation increment is achieved. Such a specific breakage state can be expressed in closed form, as follows:

A1 $$\bar{B}=\frac{1}{2}\left[\left(\frac{2}{\theta }-c_{\alpha }\right)-\sqrt{{\left(\frac{2}{\theta }-2\right)}^{2}+c_{\alpha }^{2}}\right].$$

By incorporating equation (A1) into (5.18), it is possible to recover an implicit expression for $c_{\alpha }^{\text{min}}$.

The identification of the corresponding minima for $c_{\phi }$ and $c_{\eta }$ is less straightforward, in that a general closed-form expression for $\bar{B}$ cannot be recovered. However, a reasonable approximation can be obtained for the particular case $c_{\phi }=c_{\eta }$. Under such circumstances, it is possible to show that equation (A1) is valid also for the flatness-elongation model, provided that $c_{\alpha }$ is replaced with $c_{\phi }=c_{\eta }$. It is therefore possible to incorporate equation (A1) into equation (6.15) to estimate the lower limit for the two evolution coefficient, which is represented in figure 8 as an L-shaped band located at the bottom left corner of the plane of the coevolution constants $c_{\phi }$ and $c_{\eta }$. Despite the limitations of these procedures, in particular with reference to multi-variable models, it is worth noting that in all of the examined circumstances of reported particle shape evolution, the optimal values of coevolution constants are well above the minimum limits, thus making the upper bound of these variables the most significant limitation in the assessment of the model constants.
